# Surgical repair of platypnea-orthodeoxia syndrome caused by patent foramen ovale, ascending aortic dilation, and pectus excavatum: a case report

**DOI:** 10.1186/s44215-023-00083-w

**Published:** 2023-08-03

**Authors:** Yuki Wada, Jun Hirokami, Atsushi Nagasawa, Akira Marui, Nobuhisa Ohno

**Affiliations:** 1 Department of Cardiovascular Surgery, Kokura Memorial Hospital, Kitakyushu, Japan; 2 Department of Cardiovascular Medicine, Kokura Memorial Hospital, Kitakyushu, Japan

**Keywords:** Platypnea-orthodeoxia syndrome (POS), Patent foramen ovale (PFO), Ascending aortic dilation, Prosthetic aortic graft, Pectus excavatum

## Abstract

**Background:**

Platypnea-orthodeoxia syndrome (POS) is a relatively uncommon clinical syndrome characterized by dyspnea and deoxygenation accompanying postural changes, such as sitting or standing from a recumbent position.

**Case presentation:**

We describe the case of a 76-year-old woman who was hospitalized with dyspnea in the sitting position, which was relieved in the right lateral decubitus position. The patient was diagnosed with POS caused by a patent foramen ovale (PFO), dilation and elongation of the ascending aorta, and pectus excavatum. We performed PFO closure and replacement of the ascending aorta via median sternotomy. The patient’s postoperative percutaneous oxygen saturation dramatically increased in the sitting position.

**Conclusions:**

Elongated and dilated ascending aorta and pectus excavatum with PFO may be risk factors for POS. In older patients, surgical intervention is an important treatment option for POS caused by cardiac and aortic comorbidities.

## Background

Platypnea-orthodeoxia syndrome (POS) is a relatively uncommon clinical syndrome characterized by dyspnea and deoxygenation accompanying postural changes, such as sitting or standing from a recumbent position. Two conditions are needed to cause this syndrome: the first is an anatomical component, such as an intra-atrial communication (e.g., patent foramen ovale [PFO], atrial septal defect [ASD]), and the second is a functional component that results in a deformation in the atrial septum (e.g., pericardial effusion, aortic aneurysm) with redirection of the shunt flow when the patient assumes an upright posture [1].

We report a case of successful surgical repair of platypnea-orthodeoxia syndrome caused by a PFO, dilation and elongation of the ascending aorta, and pectus excavatum.

## Case presentation

**Fig. 1:**
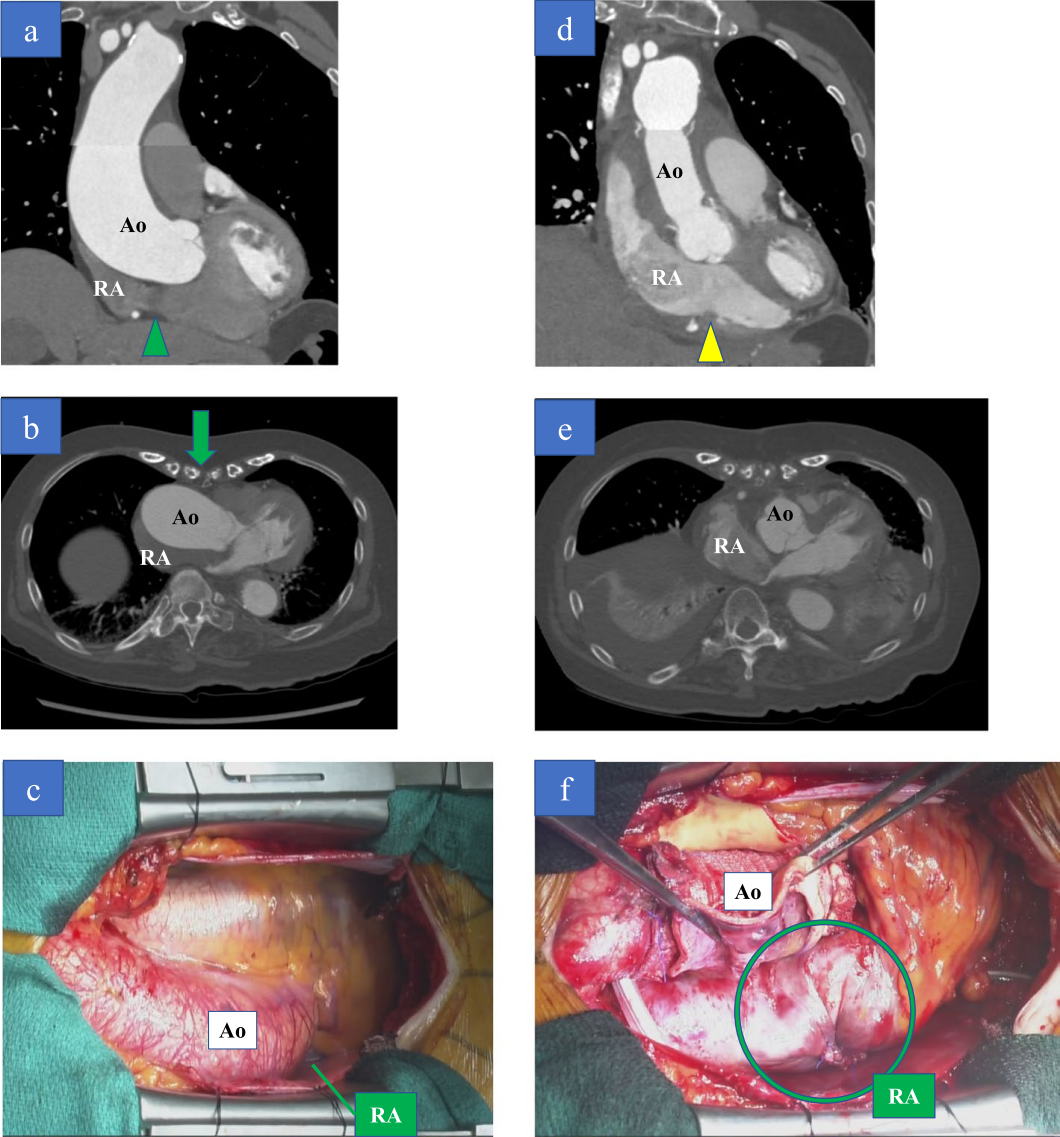
**a** and **b** Preoperative contrast-enhanced computed tomography shows deformity of the right atrium and narrowing of the tricuspid valve tract compressed by ascending aortic dilation and elongation and pectus excavatum (green arrowhead). **d** and **e** Postoperative contrast-enhanced computed tomography shows that the right atrium and tricuspid valve tract are not compressed by the ascending aorta (yellow arrowhead). **c** and **f** Before the replacement of the ascending aorta, the right atrium was compressed and hidden by a dilated and elongated ascending aorta (**c**). After the replacement of the ascending aorta, the RA is no longer compressed (**f**). Ao, Aorta; RA, right atrium

**Fig. 2:**
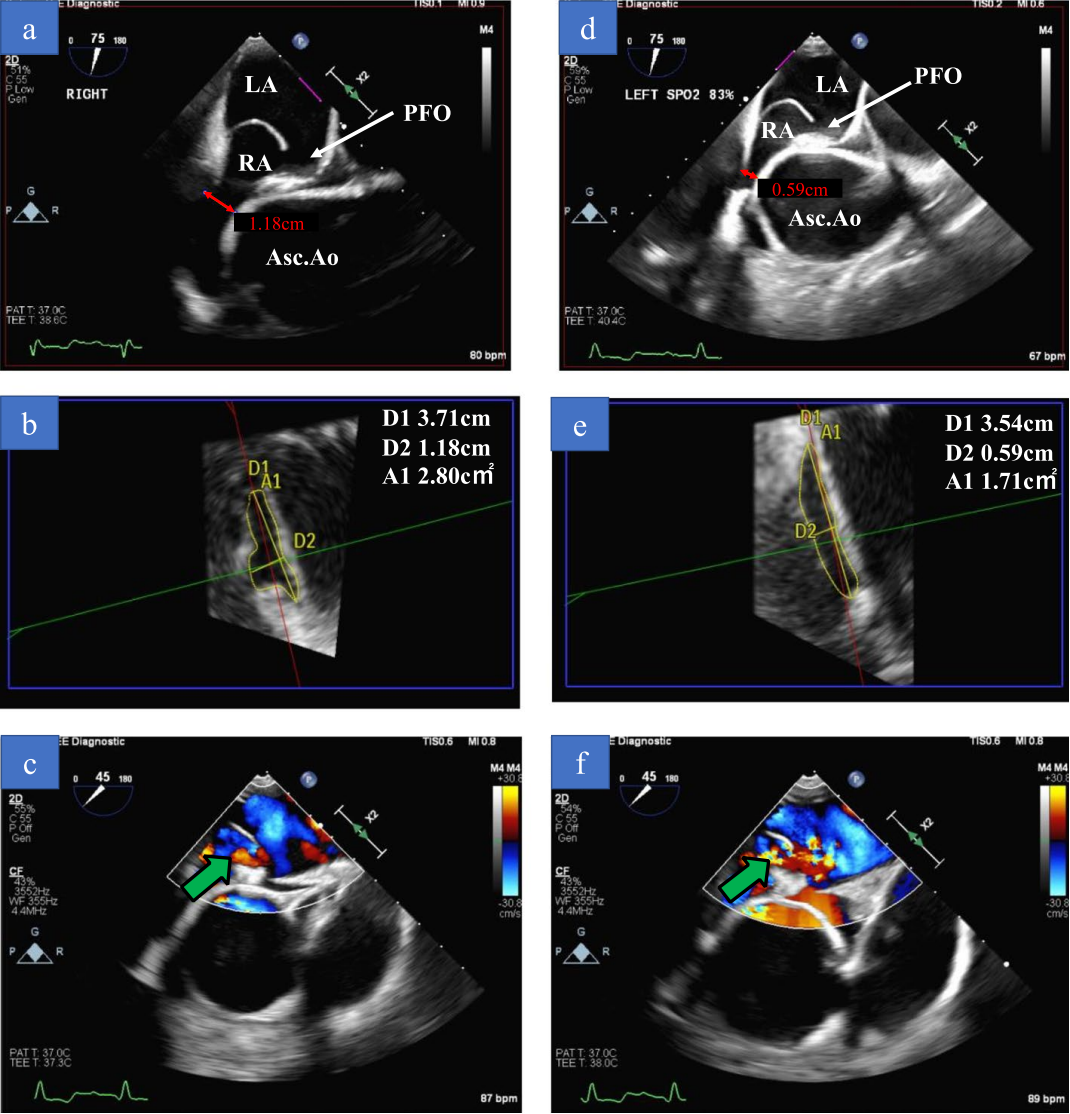
**a**–**c** RLDP. **d**–**f** Left lateral decubitus position. **a** and **d** TEE shows the elongated and dilated ascending aorta causing greater compression of the RA in the LLDP than in the RLDP. **b** and **e** Transaxial plane of the RA by TEE showing greater compression of the RA in the LLDP (area 1.71 cm^2^) than in the RLDP (2.80 cm^2^). **c** and **d** Shunt flow is enhanced by postural changes from the RLDP to the LLDP. Asc. Ao, ascending aorta; LA, left atrium; RA, right atrium; PFO, patent foramen ovale; RLDP, right lateral decubitus position; TEE, transesophageal echocardiography

A 76-year-old woman was referred to our hospital with complaints of dyspnea in the sitting position, which was relieved in the right lateral decubitus position (RLDP). Her medical history included hypertension, and she had a significant family history as her brother suffered a ruptured aortic aneurysm. Her height was 1.45 m, and her weight was 47.9 kg. Her percutaneous oxygen saturation (SpO_2_), blood pressure, and pulse rate were 88% (room air), 172/111 mmHg, and 80/min, respectively. There was no cardiac murmur, and her lungs were clear on auscultation. There was no peripheral edema. Her SpO_2_ decreased from 97% (reservoir mask, 10 L/min) in the RLDP to 75% (reservoir mask, 10 L/min) in the supine and the left decubitus position (LLDP) and 72% (reservoir mask, 10 L/min) in the sitting position. Transthoracic echocardiography showed preserved left ventricular ejection fraction and a positive microbubble test result. Contrast-enhanced computed tomography (CT) revealed no pulmonary disease but showed a deformity of the right atrium (RA) and narrowing of the tricuspid valve tract compressed by ascending aortic dilation (50 mm in diameter) (Fig. 1a, b), ascending aortic elongation (Fig. 1a), and pectus excavatum (Fig. 1b). Transesophageal echocardiography revealed a large PFO (15.6 mm in diameter) (Fig. 2a, d). The elongated and dilated ascending aorta caused a greater degree of compression of the RA in the *LLDP* than in the RLDP (Fig. 2a, b, d, and e). The shunt flow was enhanced by a postural change from the RLDP to the LLDP (Fig. 2c, f). Right heart catheterization was performed, and the mean pulmonary artery pressure and mean right atrial pressure were both 8 mmHg. The catheterization revealed no pulmonary hypertension. The patient was diagnosed with POS with a right-to-left shunt through the PFO caused by a deformity of the RA, narrowing and compression of the tricuspid valve tract by a dilated and elongated ascending aorta, and pectus excavatum. Transcatheter closure of the PFO was considered; however, it was not suitable given the RA deformity. Surgical treatment was planned for the PFO and ascending aortic dilation and elongation, but her pectus excavatum was mild, with no indication for thoracoplasty.

A median sternotomy was performed under general anesthesia. The patient’s RA was compressed and hidden by the dilated and elongated ascending aorta (Fig. 1c). Cardiopulmonary bypass (CPB) was established with ascending aortic and bicaval cannulation, with tourniquets secured around them. The aorta was cross-clamped, and myocardial protection was achieved with intermittent blood cardioplegia. The PFO was directly closed via a right atriotomy. The ascending aorta was replaced with a prosthetic graft (J graft 26 mm, Japan Lifeline, Tokyo, Japan). After the replacement of the ascending aorta, the right atrium was no longer compressed (Fig. 1f). The aortic cross-clamp and total CPB times were 83 and 134 min, respectively.

The patient’s postoperative SpO_2_ was 97% (room air) in the sitting position. Contrast-enhanced CT performed on postoperative day 4 revealed that the RA and tricuspid valve tract were no longer compressed by the ascending aorta (Fig. 1d). No adverse events occurred during or after the surgery.

## Discussion and conclusions

POS is a rare clinical syndrome characterized by dyspnea and deoxygenation accompanying postural changes, such as sitting or standing from a recumbent position [1]. The first case of POS was reported by Burchell et al. in 1949 [2]. Two etiological components are needed to cause POS: an anatomical component in the form of interatrial communication and a functional component that produces a deformity in the atrial septum and results in a redirection of shunt flow when the patient assumes an upright posture. The anatomical component is an ASD, PFO, or fenestrated atrial septal aneurysm. The functional components are (1) cardiac (e.g., pericardial effusion, constrictive pericarditis), (2) pulmonary (e.g., emphysema, arteriovenous malformation, pneumonectomy, amiodarone toxicity), (3) abdominal (e.g., liver cirrhosis, ileus), and (4) vascular (e.g., aortic aneurysm, aortic elongation) factors [3]. Thoracic deformities (e.g., severe kyphosis, pectus excavatum) can also cause POS [4, 5]. In this patient, the anatomical component was PFO; the functional components were dilation and elongation of the ascending aorta and pectus excavatum. She became symptomatic at an advanced age, probably because of the aortic dilation and elongation, which are functional components of POS, resulting from longstanding hypertension, age, and family history.

Definitive management of POS with a PFO or ASD involves closure of the shunt, and transcatheter closure of the PFO or ASD is currently available [6]. In this patient, closure of the PFO was considered but was not feasible owing to her cardiac anatomy (deformity of the RA). Surgical repair of the POS has also been reported. Soga et al. and Takashima et al. discussed a successful surgical repair of POS by shortening the elongated ascending aorta [7, 8]. In this patient, the ascending aorta was not only elongated but also dilated. According to the JCS/JSCVS/JATS/JSVS 2020 Guideline, among patients with tricuspid aortic valve with an ascending aortic diameter > 5.5 cm, prophylactic aortic surgery is recommended for those with noninherited aortic aneurysm. However, absolute aortic diameter cutoffs have inherent limitations, including the inability to account for height and sex. Several studies have recently addressed the association between height and the ascending aortic diameter. Zafar et al. reported that patients with a height-based aortic height index (aortic diameter (cm)/height (m)) of 3.21–4.06 cm/m are at high risk of developing ascending aortic complications [9]. Masri et al. discussed that the ascending aortic area (cm^2^)/height (m) ratio was associated with a higher risk of long-term mortality, whereas aortic surgery was associated with significantly improved long-term survival [10]. In this case, the patient had a low height (1.45 m), and the ascending aortic diameter was 50 mm. The height-based aortic height index was 3.45 cm/m, and the ascending aortic/height ratio was 13.5 cm^2^/m. Furthermore, she had a family history of ruptured aortic aneurysm. We assessed that the patient had a high risk of ascending aortic complication, and thus, ascending aortic replacement was indicated. Therefore, we replaced the ascending aorta with a prosthetic graft and performed PFO closure rather than shortening the ascending aorta and PFO closure.

POS is a rare cause of dyspnea; however, many patients are not correctly diagnosed. Elongated and dilated ascending aorta and pectus excavatum with PFO may be risk factors for POS. Transcatheter closure of the PFO is sometimes difficult in older adult patients owing to cardiac and aortic comorbidities. Surgical intervention is an important treatment option for POS caused by cardiac and aortic comorbidities.

## Data Availability

Data sharing is not applicable to this article as no datasets were generated or analyzed during the current study.

## Abbreviations

ASD: Atrial septal defect
CPB: Cardiopulmonary bypass
CT: Computed tomography
LLDP: Left lateral decubitus position
PFO: Patent foramen ovale
POS: Platypnea-orthodeoxia syndrome
RA: Right atrium
RLDP: Right lateral decubitus position
SpO_2_: Percutaneous oxygen saturation
